# P2X7 Receptor Indirectly Regulates the JAM-A Protein Content via Modulation of GSK-3β

**DOI:** 10.3390/ijms20092298

**Published:** 2019-05-09

**Authors:** Karl-Philipp Wesslau, Anabel Stein, Michael Kasper, Kathrin Barth

**Affiliations:** Institute of Anatomy, Dresden University of Technology, D-01307 Dresden, Germany; karl-philipp@wesslau.de (K-P.W.); bella.stein@yahoo.de (A.S.); michael.kasper@tu-dresden.de (M.K.)

**Keywords:** JAM-A, P2X7 receptor, mouse lung, alveolar epithelium, bleomycin-induced lung injury, GSK-3β

## Abstract

The alveolar epithelial cells represent an important part of the alveolar barrier, which is maintained by tight junction proteins, particularly JAM-A, occludin, and claudin-18, which regulate paracellular permeability. In this study, we report on a strong increase in epithelial JAM-A expression in P2X7 receptor knockout mice when compared to the wildtype. Precision-cut lung slices of wildtype and knockout lungs and immortal epithelial lung E10 cells were treated with bleomycin, the P2X7 receptor inhibitor oxATP, and the agonist BzATP, respectively, to evaluate early changes in JAM-A expression. Biochemical and immunohistochemical data showed evidence for P2X7 receptor-dependent JAM-A expression in vitro. Inhibition of the P2X7 receptor using oxATP increased JAM-A, whereas activation of the receptor decreased the JAM-A protein level. In order to examine the role of GSK-3β in the expression of JAM-A in alveolar epithelial cells, we used lithium chloride for GSK-3β inhibiting experiments, which showed a modulating effect on bleomycin-induced alterations in JAM-A levels. Our data suggest that an increased constitutive JAM-A protein level in P2X7 receptor knockout mice may have a protective effect against bleomycin-induced lung injury. Bleomycin-treated precision-cut lung slices from P2X7 receptor knockout mice responded with a lower increase in mRNA expression of JAM-A than bleomycin-treated precision-cut lung slices from wildtype mice.

## 1. Introduction

Alveolar epithelial cells (AEC) represent the most vulnerable cells of the distal lung parenchyma and consist of flat AECI type I and cuboidal AECII type II cells in most vertebrates, including humans. Under normal physiological conditions, both cell types are involved in gas exchange and fluid homeostasis [1] and participate in alveolar barrier functions and wound repair processes after lung injury [2]. To maintain cellular polarity and barrier functions of AEC, several types of intercellular junctions exist such as tight junctions (TJ), adherens junctions (AJ), gap junctions, and desmosomes. TJ proteins are particularly important in the regulation of the transcellular permeability of AEC.

Besides their completely different morphological appearance, AECI and AECII specifically differ in their protein pattern, which allows, to a certain degree, their distinction from each other [3]. Knockout of AECI-specific proteins lead to the early death of the animals (T1α knockout, [4], or the lungs exhibit a pathologic phenotype (e.g., caveolin-1 [5], RAGE [6], and aquaporin-5 [7] knockouts). One exception is the P2X7 receptor deficient mouse, which does not exhibit a single sign of histomorphological alterations during its lifetime [8]. The P2X7 receptor (P2X7R) is a ligand-gated ion channel activated by extracellular ATP. In the most distal part of lung, P2X7R is selectively present in AECI [9] and in alveolar macrophages [10]. The intracellular pathways activated by the receptor influence pulmonary inflammation (reviewed in Reference [11]) and the P2X7R knockout (P2X7^−/−^) lung show altered tight junction protein expression [12].

In experimental studies, P2X7^−/−^ deficient mice have presented dramatically reduced lung inflammation with reduced fibrosis markers in the bleomycin (BLM) model [13]. Deletion of P2X7R has a protective effect and the receptor is a therapeutic target for the amelioration of hyperoxia-induced lung injury [14]. P2X7^−/−^ animals showed no significant effect of LPS on lung function, alveolar collapse, or fiber deposition in lung parenchyma when compared with wildtype (WT) mice [15].

Treatment with BLM, an anti-cancer agent, is often used as an experimental model of lung injury and pulmonary fibrosis. Molecular changes after BLM exposure include genes encoding growth factors, signaling molecules, and structural proteins, for example, caveolin-1 and diverse junctional proteins. BLM causes an increase in reactive oxygen species and thus induces apoptosis in epithelial and other cells of the lung, leading to disruption of the alveolar barrier. Recently, it was shown that bleomycin-induced lung injury was attenuated in P2X7^−/−^ mice [13].

The TJ and AJ are collectively referred to as the apical junctional complex (AJC) and constitute apical intercellular contacts. The AJC contains the key transmembrane proteins occludin, the claudin protein family, and junctional adhesion molecules (JAM) localized to the TJ, as well as E-cadherin in the AJ. JAMs are expressed by a variety of different cells, mainly epithelial cells, endothelial cells, and cells of the immune system, e.g., leukocytes. The importance of JAM-A in regulating barrier function is shown for JAM-A in epithelial and endothelial cells where siRNA mediated loss of JAM-A expression results in enhanced permeability, as determined by transepithelial resistance (TER) [16]. A complex series of poorly understood signaling events establish epithelial barrier function culminating in the formation of mature TJs, whereby JAM-A seems to be important in early events required for TJ assembly. Adhesion complexes are not formed at low Ca^2+^ concentration in epithelial cells.

In earlier work, we have shown that high intracellular Ca^2+^ content through activation of P2X7R after BLM treatment leads to increased protein kinase (PKC)-β1 in alveolar epithelial cells [17]. The comparison of lung tissues from WT and P2X7^−/−^ mice revealed decreased protein and mRNA levels of PKC-β1 and calmodulin (CaM). We demonstrated that the inhibition of P2X7R after BLM treatment also leads to decreased CaM and PKC-β1 content. This indicates that in the BLM model, P2X7R is involved in the regulation of intracellular calcium content and that the PKC-β1 acts downstream of the P2X7R. By stimulating and inhibiting various isoforms, the conventional PKCs, including the isoforms α, β1, β2, and γ are activated by calcium and diacylglycerol. Both factors have been described as triggers of TJ dissolution [18]. In addition to modified TJ protein levels in the P2X7^−/−^, we also found an increased inactivation of the glycogen synthase kinase (GSK)-3β in the P2X7^−/−^ compared to the WT mice [12]. In vitro experiments demonstrated that GSK-3β phosphorylation mediated by PKC enhanced GSK-3β activity. It has also been reported that in vitro GSK-3β is inactivated in the same manner by particular forms of PKCs [19]. The physiological importance of GSK-3β activity in the regulation of the normal epithelial barrier was shown by Severson et al. [20], which implicates the active role of GSK-3β in controlling the expression of the AJC proteins occludin, claudin-1, and E-cadherin. 

The aim of this study was to investigate the expression of the tight junction molecule JAM-A in WT and P2X7^−/−^ mice and to investigate the involvement of GSK-3β, which has previously been shown to be increased in P2X7^−/−^ mice [12]. We further studied the influence of BLM on JAM-A in precision-cut lung slices (PCLS) of WT and P2X7^−/−^ mice and in immortal AECI-like E10 cells and whether the GSK-3β(Ser9) phosphorylation changed after BLM treatment. The influence of the inhibition of P2X7R under BLM treatment was studied using the P2X7R inhibitor oxATP. We also investigated whether inactivation of P2X7R led to changes in the phosphorylation of GSK-3β at Ser9 and if this subsequently had an impact on JAM-A protein content.

## 2. Results

### 2.1. P2X7^−/−^ Mice Show Strongly Enhanced JAM-A Protein Level in the Lung Parenchyma

**Figure 1:**
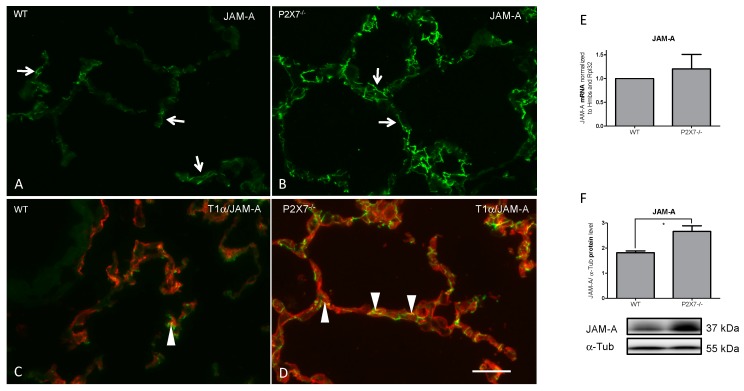
Frozen sections of mouse lung tissue. Immunofluorescence demonstration of JAM-A in WT and P2X7^−/−^ mice (**A**–**D**). Note the increase in immunoreactivity of JAM-A in P2X7^−/−^ mice (**B**,**D**). (**C**,**D**) Double immunofluorescence with the AECI marker T1α (TexasRed). Arrows show the linear pattern of JAM-A. Arrowheads depict examples of epithelial junctions. Bar = 100 µm. Corresponding mRNA (**E**) (Wildtype (WT) normalized to 1; *n* = 3; *p*-value 0.5737) and protein (**F**) (*n* = 3, *p*-value 0.0357) levels in lung homogenates. * *p* < 0.05.

We analyzed the mRNA expression and total protein content of JAM-A in lung tissue homogenates of WT and P2X7^−/−^ mice (Figure 1).

A significant increase in JAM-A protein content was found in the P2X7^−/−^ mice. The total mRNA level of JAM-A was also found to be increased.

Immunofluorescence staining confirmed the change in JAM-A expression. WT mice showed a predominantly linear staining pattern of localization for JAM-A. This pattern of immunoreactivity did not change in the P2X7^−/−^ mice. Only quantitative alterations were observable. There were no signs of disrupted junctions. Double immunofluorescence experiments with AECI specific T1α revealed a prominent JAM-A localization related to the AECI-AECI and AECI-AECII border. 

### 2.2. Influence of BLM Treatment on mRNA Expression of JAM-A and Localization in the Lung Tissue of P2X7^−/−^ Mice in Comparison to the WT

**Figure 2:**
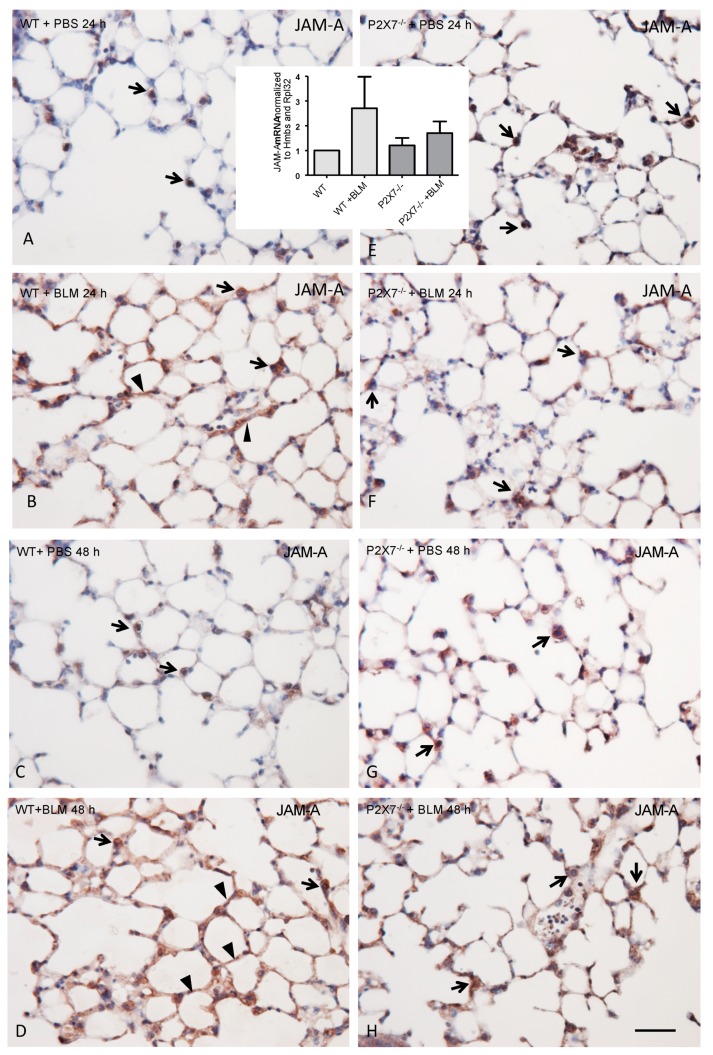
Paraffin sections from embedded PCLS after 300 mU/mL BLM exposure for 24 h (**A**,**B**,**E**,**F**) and 48 h (**C**,**D**,**G**,**H**). Immunoperoxidase demonstration of JAM-A in WT (**A**–**D**) and P2X7^−/−^ (**E**–**H**) mice. Note the preferable immunostaining of AECII in untreated WT (arrows in **A**,**C**), a weak increase in P2X7^−/−^ mice (**E**,**G**), and the strongest immunostaining of the AECs in the BLM-treated WT mice (**B**,**D**). Arrowheads depict the alveolar lining of JAM-A immunoreactivity. Bar = 100 µm. Inset over (**A**) and (**E**): Analysis of mRNA content in paraffin sections of PCLS from WT and P2X7^−/−^ mice after 24 h. mRNA content of *JAM-A* was analyzed by quantitative real time RT-PCR using *Hmbs* and *Rpl32* as housekeeping genes. Charts are represented as mean ± SEM (WT normalized to 1; *n* = 3; *p*-value 0.4550).

To investigate whether the BLM treatment in the lung tissue of P2X7^−/−^ led to an altered expression of JAM-A in comparison to the WT animals, the PCLS of wildtype and P2X7^−/−^ mice were prepared and treated with BLM for 24 h and 48 h (Figure 2). Using quantitative RT-PCR, we were able to demonstrate a marked increase in JAM-A expression in the PCLS of BLM-treated WT mice compared to BLM-treated PCLS of P2X7^−/−^ mice (Figure 2, inset in A and E).

Immunoperoxidase staining for JAM-A in the PCLS of WT lungs exhibited prominent staining at the AECI/II border and some additional cytoplasmic AECII staining, with increased immunoreactivity in the PCLS of P2X7^−/−^. The entire JAM-A immunoreactivity, as seen in frozen sections (compared to Figure 1), could not be reproduced in the paraffin sections, since fixation and paraffin embedding impaired the immunoreactivity and the structural conciseness of immunolocalization. The 24 h treatment with BLM led to a strongly enhanced immunoreactivity of the entire alveolar lining layer in the WT, but to a lesser extent in the P2X7^−/−^ (Figure 2). This effect was stronger after 48 h of BLM treatment (Figure 2).

Some additional endothelial JAM-A immunostaining could not be excluded, since double staining with endothelial markers could not be performed in the present study due to the lack of suitable antibodies.

### 2.3. The Inhibition of GSK-3β Leads to the Reduction of the Protein Content of JAM-A under BLM Treatment

**Figure 3:**
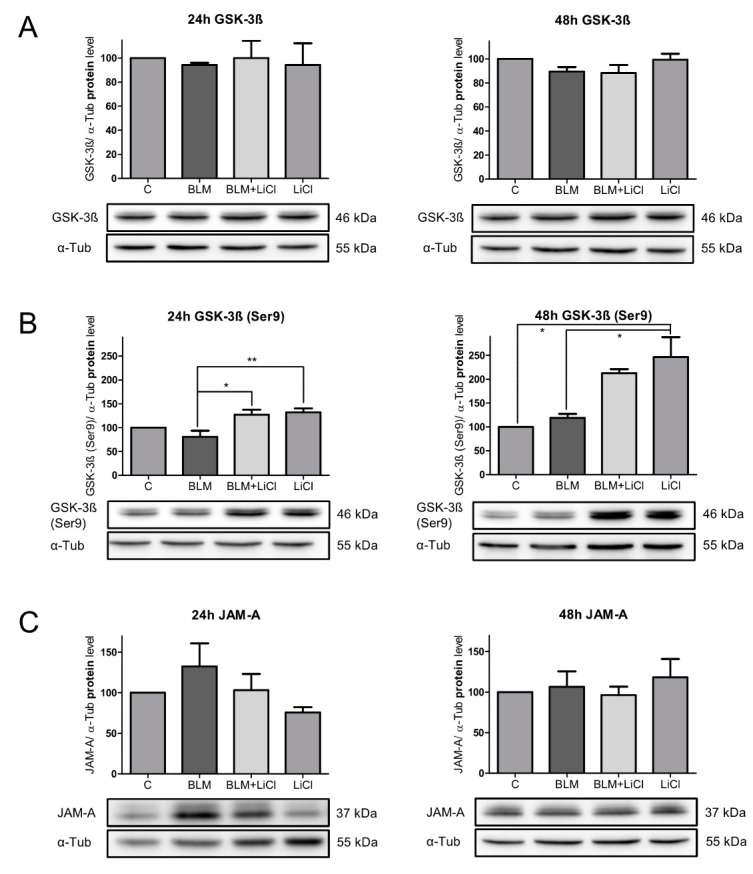
Expression of total GSK-3β (**A**), GSK-3β(Ser9) (**B**), and JAM-A (**C**) were analyzed by Western blot after 24 h and 48 h treatment with 100 mU/mL BLM, 100 mU/mL BLM, and 10 mM LiCl or 10 mM LiCl alone. Equal protein amounts of cell lysates were used in SDS-PAGE and analyzed by Western blot. α-Tub served as the loading control. Untreated cells were used as the control and normalized to 100%. Representative blots from three independent experiments are shown. Charts presented as mean ± SEM (*n* = 3) of GSK-3β /α-Tub, GSK-3β(Ser9)/α-Tub, and JAM-A/α-Tub. *P*-values: 24 h GSK-3β 0.9447; 48 h GSK-3β 0.0625; 24 h GSK-3β(Ser9) 0.0046; 48 h GSK-3β(Ser9) 0.0066; 24 h JAM-A 0.186; and 48 h JAM-A 0.6123. * *p* < 0.05, ** *p* < 0.01.

In the following experiment, different influences on JAM-A were evaluated. Based on the findings in the P2X7^−/−^ mice where the inactivated form of GSK-3β, the GSK-3β(Ser9), was upregulated compared to WT [12], the GSK-3β(Ser9) protein level was investigated after BLM treatment in the alveolar epithelial E10 cells. In order to examine the role of GSK-3β in the expression of JAM-A, we used lithium chloride (LiCl), an inhibitor of GSK-3β, to treat the undamaged cells. Additionally, inactivation of GSK-3β under BLM treatment was performed to investigate the effects of inactivated GSK-3β on JAM-A under these conditions. After 24 h and 48 h of treatment with BLM, BLM + LiCl, or LiCl alone, the expression of the total GSK-3β was unchanged under all conditions when compared with the untreated E10 cells (Figure 3A).

After 24 h of BLM treatment, no increase in GSK-3β(Ser9) was seen (Figure 3B). The addition of LiCl to BLM or LiCl alone increased the expression of GSK-3β(Ser9) only slightly (Figure 3B), but significantly in comparison to the BLM-treated cells.

As shown in Figure 3C, 24 h of BLM treatment of the E10 cells induced an increase of 132.3% in JAM-A. Inhibition of GSK-3β with LiCl in combination with BLM reduced the increase of JAM-A, indicating that the inactivation of the GSK-3β under BLM treatment had consequences for the expression of JAM-A (Figure 3C). Treatment of E10 cells with LiCl alone also downregulated the protein content of JAM-A to 75.6% compared to the untreated control cells.

After 48 h of BLM treatment, no increase in GSK-3β(Ser9) was also seen (Figure 3B). The addition of LiCl after 48 h of BLM treatment resulted in a strong increase in the protein content of the inactive form GSK-3β(Ser9) to 178.5% compared to the BLM-treated cells (Figure 3B). Likewise, treatment with LiCl alone led to a dramatic increase in GSK-3β(Ser9).

After 48 h of BLM treatment, the early increase in JAM-A returned to the level seen in the control cells (Figure 3C) The strong upregulation of GSK-3β(Ser9) under BLM treatment or by sole LiCl treatment in the control cells did not lead to any changes in the JAM-A protein.

In summary, early BLM treatment (24 h) in alveolar epithelial cells induced an increase in JAM-A. The BLM treatment did not inactivate GSK-3β within a period of 48 h. The early rise of JAM-A after BLM exposure could be reduced to the protein level of the control cells by inactivation of GSK-3β. This means that the upregulation of the inactive form of GSK-3β prevents the rise of JAM-A under BLM treatment.

### 2.4. The P2X7R Indirectly Regulates JAM-A Protein Content by the Modulation of GSK-3β(Ser9)

The aim of the following experiment in the E10 cells was to investigate how the inhibition of P2X7R under BLM treatment (24 h) affected the inactive form of GSK-3β and whether subsequent changes in JAM-A occurred. The increase in the protein content of P2X7R after BLM treatment in the E10 cells has already been shown in one of our previous studies [17]. Additionally, the increase in JAM-A after BLM treatment was confirmed (Figure 3) and there was no inactivation of GSK-3β at this time when compared to the untreated control cells (Figure 3).

**Figure 4:**
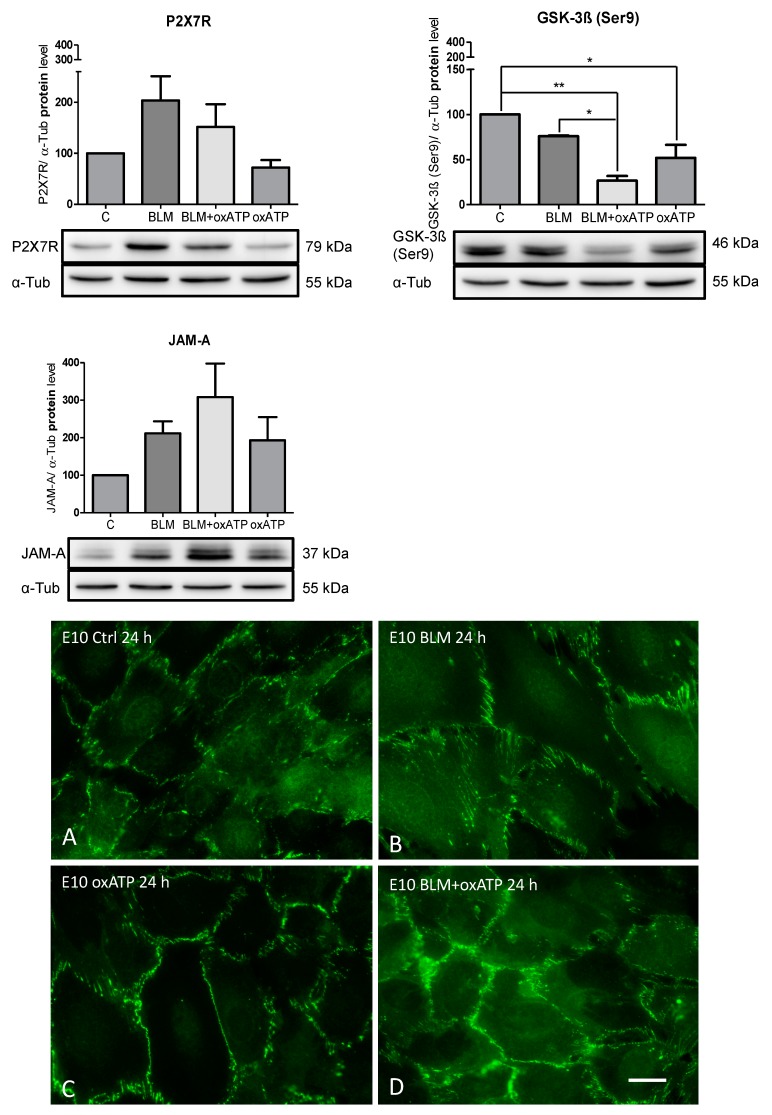
Effects of P2X7R inhibition by 150 µM oxATP, which was added 2 h prior to 100 mU/mL BLM treatment. Equal protein amounts of cell lysates were used in SDS-PAGE and analyzed by Western blot. α-Tub served as the loading control. Untreated cells were used as the control and normalized to 100%. Representative blots from three independent experiments are shown. Charts are presented as the mean ± SEM (*n* = 3) of P2X7R/ α-Tub, GSK-3β(Ser9)/ α-Tub and JAM-A/ α-Tub. P-values: P2X7R 0.0338; GSK-3β(Ser9) 0.002; and JAM-A 0.05. Immunofluorescence demonstration of JAM-A in untreated (**A**), BLM (**B**), or oxATP (**C**) treated E10 cells. Note the increased cell size after BLM exposure (**B**), which was ameliorated after oxATP (**D**). Representative images of multiple experiments (*n* = 3) are shown. Bar = 20 µm. * *p* < 0.05, ** *p* < 0.01.

To test the hypothesis that P2X7R could indirectly modulate JAM-A following BLM treatment by regulating the inactive form of GSK-3β, we studied the GSK-3β(Ser9) and JAM-A protein contents after inhibition of P2X7R by oxATP (Figure 4).

The inhibition of P2X7R under BLM treatment led to a strong decrease in the GSK-3β(Ser9) protein content in the alveolar epithelial cells in comparison to the untreated or BLM-treated cells. The significant reduction in GSK-3β(Ser9) protein content compared to BLM-treated cells resulted in a re-upregulation of JAM-A. Treatment with oxATP alone led to significant downregulation of GSK-3β(Ser9) when compared to the untreated cells. The inhibition of P2X7R under these conditions also produced an increase in JAM-A when compared to the untreated cells.

### 2.5. Localization of JAM-A in Alveolar Epithelial Cells after BLM Treatment and the Influence of oxATP

Confluent E10 cells were incubated with 100 mU/mL BLM to study the effect of BLM on the distribution of JAM-A. Immunofluorescence revealed a regular localization of JAM-A to TJ at sites of cell–cell contact in the control cells (Figure 4). BLM exposure for 24 h (Figure 4B) or oxATP alone (Figure 4C) did not change JAM-A immunoreactivity and its cellular localization, but the cell size increased after BLM exposure. oxATP and BLM together normalized the BLM induced cell swelling (Figure 4D).

### 2.6. The P2X7R Agonist BzATP Affects the JAM-A Protein Content

**Figure 5:**
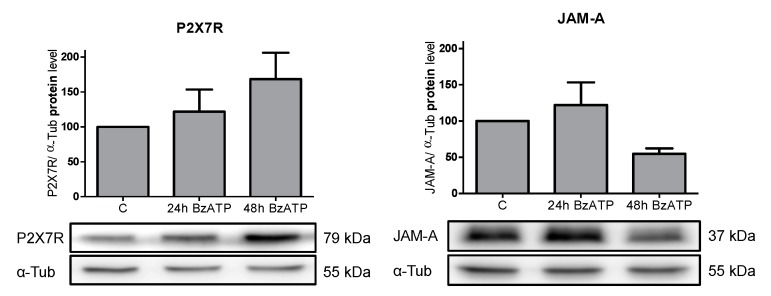
Analysis of protein levels in alveolar epithelial cell line E10 after treatment with 150 µM BzATP. Cells were treated with BzATP for 24 h and 48 h. For SDS-PAGE, equal protein amounts of cell lysates were used and analyzed by Western blot with antibodies against P2X7R, JAM-A, and α-Tub. Untreated cells were used as the control and normalized to 100%. Protein levels were normalized to α-Tub and are shown as the mean ± SEM (*n* = 3) in relation to the control. One representative blot is pictured. P-values: P2X7R 0.1667 and JAM-A 0.1048.

Next, we explored whether stimulation of P2X7R in alveolar epithelial E10 cells with BzATP would induce a modulation in the JAM-A protein content. E10 cells were exposed to 100 μM BzATP for one and two days to stimulate P2X7R. The Western blots are shown in Figure 5.

The response to purinergic receptor stimulation by BzATP, which presumably involves a Ca^2+^ channel opening, resulted in an increase in Ca^2+^ [17]. As an indication for this, we were able to show in previous work that the CaM content was increased after BzATP treatment [17]. In the present experiments, we demonstrated that the JAM-A protein content was influenced by the BzATP treatment. After 24 h of BzATP treatment in E10 cells, a minimal increase in the JAM-A protein concentration occurred, whereas a stronger decrease was seen after 48 h (Figure 5).

## 3. Discussion

Maintenance of the integrity of the alveolar barrier is realized by TJ among neighboring AECs consisting of occludin, claudins, ZOs, and JAM-A.

Recently, we have demonstrated that in P2X7^−/−^ mice, claudin-18 is upregulated and the inactive form of GSK-3β, GSK-3β(Ser9), is also upregulated in comparison to WT mice [12]. Our current data show that another TJ protein, JAM-A, in P2X7^−/−^ mice is strongly upregulated at the protein level. P2X7^−/−^ mice exhibited reduced lung inflammation with reduced fibrosis markers such as lung collagen [13]. Deletion of P2X7R is a protective factor in acute lung injury [14]. Through the upregulation of JAM-A and claudin-4 and -15 in a kinase-dependent manner, an improved barrier function of the oral epithelium could be demonstrated [21].

Our data suggest that increased constitutive JAM-A protein level may have a protective effect against BLM-induced lung injury in P2X7^−/−^ mice. BLM-treated PCLS from P2X7^−/−^ mice responded with a slighter increase in mRNA expression of JAM-A than BLM-treated PCLS from WT mice. The reduced level of JAM-A upregulation in the P2X7^−/−^ mice in comparison to the WT mice indicates a lower sensitivity of this protein to the effects of BLM in the alveolar epithelium.

JAM-A is concentrated at epithelial and endothelial tight junctions and it has been shown that JAM-A localizes to claudin-based tight junction fibrils in epithelial cells [22]. It does not directly regulate the barrier between the cells, but rather interacts as a signaling molecule with divergent downstream target proteins [23]. Nevertheless, in various epithelial and endothelial cell lines including primary rat alveolar epithelial cells, it has been shown that siRNA mediated downregulation of JAM-A expression results in enhanced paracellular permeability, as determined by TER measurements [16]. Long-term treatment of PCLS (five days) with BLM showed a strong downregulation of JAM-A when compared to the untreated PCLS (own unpublished data).

To examine the consequences of decreased GSK-3β activity on JAM-A under BLM treatment, the GSK-3β inhibitor LiCl was used in this study. JAM-A is affected by the inhibition of GSK-3β. The initial upregulation of JAM-A under BLM treatment is prevented by the inactivation of GSK-3β. Inhibition of GSK-3β causes JAM-A expression to remain at levels comparable to that of the untreated cells.

This result indicates that inhibition of GSK-3β has a positive effect on the deregulatory changes in JAM-A expression under BLM treatment. Several studies have shown that the inhibition of GSK-3β reduces the development of acute lung injury and inflammation and has a protective effect on lung fibrosis induced by BLM [24,25]. The P2X7^−/−^ mice, which showed no fibrotic changes under BLM treatment, had a high constitutive expression of GSK-3β(Ser9) [12], which is an indication of the protective effect of GSK-3β inhibition.

While the data on GSK-3β inhibition in acute lung injury are fairly clear, there are little data on its effect on TJ proteins. Severson et al. [20] have shown that endogenous GSK-3β activity is required for maintenance of the AJC, and therefore for epithelial barrier function by regulating the expression of transmembrane proteins claudin-1 and occludin. They also observed a differential decrease in the labeling of key AJC proteins following GSK-3β inhibition, a decrease in occludin, claudin-1, and E-cadherin protein levels, but they could not show any effect on JAM-A expression and localization. They reported that in both human intestinal (SK-CO15) and kidney (MDCK) epithelial cells, a decrease in GSK-3β activity interfered with epithelial cell–cell transitions, thereby increasing paracellular permeability.

It was previously shown by Bazzoni et al. [26] that the absence of JAM-A enhanced cell motility, increased membrane protrusions, affected microtubule stability, and reduced focal adhesions in endothelial cells. The consequences of JAM-A absence were reversed on treatment with GSK-3β inhibitors.

In this study, treatment with oxATP alone reduced the expression of P2X7R and increased JAM-A when compared to the untreated WT cells. After the addition of oxATP to BLM-treated cells, the P2X7R was downregulated, but a higher amount of JAM-A protein was still measured than in the untreated cells. In this case, JAM-A was also upregulated. Furthermore, inactivation of P2X7R by oxATP led to a substantial reduction in the constitutively present level of inactive GSK-3β in untreated and BLM-treated cells.

**Figure 6:**
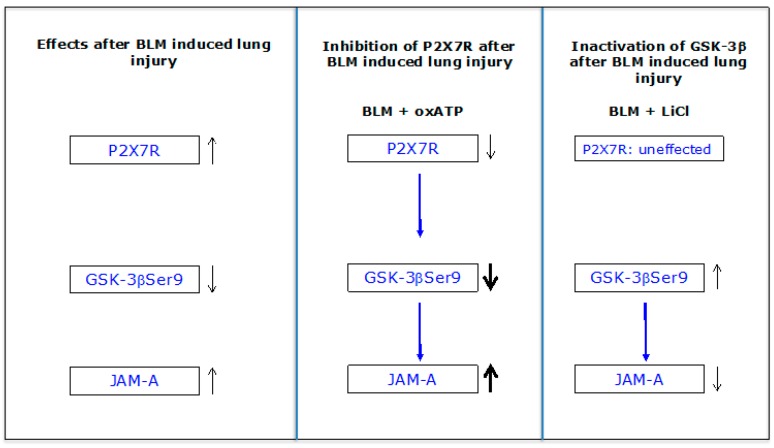
BLM treatment results in increased protein levels of P2X7R and JAM-A as well as in a reduced content of the inactive form of GSK-3β GSK-3β(Ser9). After inhibition of P2X7R by oxATP under BLM exposure, the effect on both proteins is further enhanced. Inactivating of the GSK-3β by LiCl under BLM exposure directly leads to a reduction of JAM-A. The influence of P2X7R on JAM-A is rather indirect.

The inhibition of P2X7R under BLM treatment resulted in the opposite effect on GSK-3β(Ser9) when compared to the effect of the GSK-3β inhibitor LiCl. The inactivated form of GSK-3β, the GSK-3β(Ser9) was even further downregulated, which was expressed downstream in the upregulation of JAM-A. Figure 6 summarizes the data:

Interestingly, the inhibition of P2X7R under BLM did not lead to the upregulation of GSK-3β as found in P2X7^−/−^ mice. The siRNA-mediated downregulation of P2X7R led in turn to the upregulation of GSK-3β in untreated alveolar epithelial cells [12]. The different effects on GSK-3β still have to be clarified. However, knockout or inhibition of P2X7R always leads to an increase in the JAM-A protein level, indicating a repressive effect of P2X7R on the expression of JAM-A.

Furthermore, we have shown that activation of P2X7R by BzATP resulted first in a very slight upregulation of JAM-A after 24 h, and then in a strong downregulation of the protein after 48 h in alveolar epithelial cells E10. Guo et al. [27] showed in E10 cells that activation of P2X7R by BzATP increased the phosphorylation of Y216 and decreased the phosphorylation at S9 in GSK-3β without affecting the total GSK-3β expression. This result indicates that BzATP stimulates GSK-3β activity. 

It has been demonstrated that JAM-A has the ability to promote the assembly and remodeling of alveolar epithelial tight junctions in response to acute lung injury and plays a protective role in preventing lung damage and promoting fluid clearance [28]. Identifying pathways that increase the expression and function of JAM-A in acute lung injury may identify new approaches to promote barrier function in response to inflammation and injury.

We were able to show for the first time that P2X7R plays an important role in the regulation of JAM-A in the alveolar epithelium. Downregulation of P2X7R or the absence of the protein leads to upregulation of JAM-A, possibly resulting in an increase in barrier function. Conversely, stimulation of P2X7R leads to downregulation of the protein. A modulating effect on JAM-A has been demonstrated under BLM treatment for GSK-3β.

## 4. Materials and Methods

### 4.1. Ethics Statement

All animal experiments were approved by the Ethics Committee of the Dresden University of Technology and the license for the removal of organs was provided by Landesdirektion Dresden (file no. 24-9168.24-1/2007-26; file no. 24-9168.24-1/2010-11).

### 4.2. Experimental Animals

WT mice were purchased from Charles River (C57BL/6; Charles River, Wilmington, USA) and the P2X7^−/−^ mice were obtained from Pfizer (B6.129P2-P2rx7^tm1Gab^/J; Pfizer, New York, NY, USA) [29]. Our animals were housed at the Animal Care Facility at the Medical Faculty “Carl Gustav Carus” of Dresden University of Technology and had steady free access to standard chow and water. All performed procedures were in accordance with the Technical University of Dresden Animal Care and Use Committee Guidelines. For our experiments, we examined the lung tissue of male and female mice with an age of 8 to 16 weeks.

### 4.3. Cell Line and Cell Culture

The mouse lung cell line E10 [30] was acquired by M. Williams (Pulmonary Center, Boston University School of Medicine, Boston, MA, USA). Cells were cultured in DMEM/Ham’s F12 medium (1:1) purchased by Gibco (ThermoFisher Scientific, Waltham, MA, USA), supplemented with 5% (*v*/*v*) fetal bovine serum (Biochrom AG Seromed, Berlin, Germany) and 2.5 mM L-glutamine (Merck KGaA, Darmstadt, Germany). E10 cells were seeded at a density of 1.5 × 10^4^ to 3 × 10^4^ cells/mL and passaged three times a week up to 25 passages and were grown at 37 °C in a 5% CO_2_ atmosphere up to a confluence of 60–80%.

For treatment of the cells, the medium was supplemented with 100 mU/mL BLM (Bleocell, STADA Arzneimittel AG, Bad Vilbel, Germany), 150 µM oxATP, 150 µM BzATP, and 10 mM LiCl (Sigma-Aldrich, Munich, Germany). OxATP and LiCl were added to the cells 1 h before BLM treatment.

### 4.4. Precision-Cut Lung Slices (PCLS) and Tissue Culture

To attain PCLS, we followed the procedure published by [31] with several modifications such as the process of obtaining, cutting, handling, and incubating the lung slices described in detail by [8]. After 24 h of incubation, the lung slices were homogenized for real-time reverse transcription PCR, Western blot analyses, or embedded in paraffin for immunohistochemistry.

### 4.5. RNA Isolation and Real-Time Reverse Transcription PCR (Real-Time RT PCR)

The isolation of total RNA was realized as previously described with some modification [8]. Primers applied for real-time RT PCR were: JAM-A (5´-TCCTGGGCTCTTTGGTACAAGG-3, 5´-TCCTGGGCTCTTTGGTACAAGG-3), mHmbs (5´-GCTTCGCTGCATTGCTGAAA-3´, 5´-CCAGTCAGGTACAGTTGCCC-3´), mRpl32 (5´-GCACCAGTCAGACCGATATGTG-3´, 5´-CTTCTCCGCACCCTGTTGTC-3´).

The identity of the PCR products was proven by melting point analysis. Using the ΔΔCT method at CFX Manager (Bio-Rad), the relative quantification of gene expression was performed with housekeeping genes *Hmbs* (hydroxymethylbilane synthase) and Rpl32 (ribosomal protein L32).

### 4.6. Western Blot Analysis

Total protein concentrations of the lysates of lung tissue and cells were determined by using the BCA Protein Assay Kit (ThermoFisher Scientific) according to the manufacture’s guidelines. Murine tissue protein extraction was performed as described previously in [12] by using the Precellys 24 Homogenizer (PEQLAB, Erlangen, Germany) and lysis buffer including 0.02 M Tris, pH 8.5; 0.125 M NaCl; and 1% (*v*/*v*) Triton X-100 with protease inhibitor Complete Tablets, EDTA-free (Roche, Basel, Switzerland).

Cell protein extraction was performed by applying ice cold PBS buffer without Mg^2+^ and Ca^2+^ (Biochrom AG, Berlin, Germany) supplemented with protease inhibitor Complete Tablets, EDTA-free (Roche) into T75 flasks (Greiner Bio-one GmbH, Frickenhausen, Germany). The cells were scratched from the surface, collected, and pelleted at 500× *g* for 5 min. After adding lysis buffer including 0.02 M Tris, pH 7.5; 0.14 M NaCl; 1 mM EDTA, pH 8.0; and 1% (*v*/*v*) Triton X-100 with protease inhibitor Complete Tablets, EDTA-free (Roche), the lysate was incubated 45 min at 4 °C with slight motion and centrifuged at 10.000× *g* for 20 min at 4 °C to collect the supernatant containing the protein.

For SDS-PAGE, the samples were diluted to a homogeneous total protein concentration, transferred into 6× SDS sample buffer (300 mM Tris, pH 6.8; 100 mM Dithiothreitol; 0.1% (*w*/*v*); 30% (*w*/*v*) glycerol; 10% (*w*/*v*) SDS), boiled at 95 °C for 5 min and 10–50 µg of total protein per sample were loaded on a 12% SDS-polyacrylamide gel. Western blot analysis was performed as described [32] with minor modifications. The PVDF Immobilon-P Membrane (Merck Millipore, Billerica, USA) was blocked for 1 h in TBS-T buffer (17 mM Tris, pH 7.4; 2.7 mM KCl; 137 mM NaCl; 0.2% (*v*/*v*) Tween 20) including 5% (*w*/*v*) dried non-fat powdered milk (Carl Roth GmbH, Karlsruhe, Germany) and incubated at 4 °C overnight with the following primary antibodies: polyclonal rabbit anti-human JAM-A (A302-891A), dilution 1:1000 (Bethyl Laboratories Inc.); polyclonal rabbit anti mouse P2X7R (APR-004; Alomone Labs Inc.), dilution 1:500; monoclonal mouse anti-human GSK-3β (610202; BD Biosciences), dilution 1:1000; monoclonal rabbit anti-human Phospho-GSK-3β (Ser9) (#9323; Cell Signaling Technology Inc.), dilution 1:1000; and monoclonal mouse anti -tubulin (sc-8035; Santa Cruz Biotechnology Inc.), dilution 1:1000.

The following secondary antibodies were used at room temperature for 1 h: donkey anti-rabbit IgG, HRP-linked F(ab)2 fragment (GE Healthcare, Chalfont St Giles, Buckinghamshire, UK), and horse anti-mouse IgG, HRP-linked antibody (Cell Signaling, Danvers, MA, USA). The chemiluminescent signal was detected by using Immobilon Western Chemiluminescent HRP Substrate (Merck Millipore) by following the manufacturer’s guidelines and Image Reader LAS-3000 (Fujifilm, Tokyo, Japan). Quantification was done with ImageJ 1.51 u free software (Wayne Rasband, National Institutes of Health, Bethesda, MD, USA) and each line was normalized to corresponding α-tubulin (α-Tub).

### 4.7. Immunohistochemistry

Fixation of 5 µm thin lung tissue in 4% (*v*/*v*) buffered formalin, embedding in paraffin, sectioning, and immunostaining were performed as described previously in [8,17]. The primary antibody against JAM-A was detected with a biotinylated secondary antibody, followed by incubation with the streptavidin/biotin-peroxidase complex (Vectastain Elite Kit, Serva, Heidelberg, Germany). As a negative control, the primary antibody was replaced with PBS or a non-immune serum.

### 4.8. Immunofluorescence

Immunofluorescence and double immunofluorescence staining on frozen cryostat sections of mouse lung tissue were performed, as previously described in Reference [33]. The following primary antibodies were used: polyclonal rabbit anti JAM-A and monoclonal mouse anti T1α clone E11, dilution 1:200, which was a kind gift from Dr. A. Wetterwald, Bern (Switzerland). As secondary antibodies, we used goat anti-rabbit IgG conjugated to fluorescein isothiocyanate (FITC, Dianova, Hamburg, Germany; dilution 1:100 (*v*/*v*)) and donkey anti-mouse IgG, Texas Red labelled (Dianova, Hamburg, Germany; dilution 1:100 (*v*/*v*)). Negative controls included the omission of the primary antibody. Acetone-methanol fixed monolayers of E10 cells were similarly incubated with the same dilution of the JAM-A antibody.

### 4.9. Statistical Analysis

One-way analysis of variance (ANOVA) was used to determine the results of Western blot analysis and real-time RT PCR with three or more groups, followed by the post hoc Bonferroni test in case significance was achieved. Statistical comparison of two groups was performed as a two tailed Student´s *t* test. Statistical analysis was done with GraphPad Prism 5.03 software (GraphPad Software, San Diego, CA, USA). We determined significance at * *p* = 0,05 and high significance at ** *p* = 0.01. A minimum of three independent experiments for each technique was performed.

## Abbreviations

BLM	Bleomycin
GSK-3β	Glycogen synthase kinase-3 beta
JAM-A	Junctional adhesion molecule-A
LiCl	Lithium chloride
PCLS	Precision-cut lung slices
PKC-β1	Protein kinase C-beta1
P2X7R	P2X7 receptor
WT	Wildtype
